# Supplementary material for: Registered report: The effectiveness of a Bhagavad Gita intervention to reduce psychological distress in homeless people—A randomised controlled trial
Source: PLoS One. 2024 Nov 5;19(11):e0310795. doi: 10.1371/journal.pone.0310795 (PMC11537408; doi:10.1371/journal.pone.0310795)
Supplement: S1 File — (DOC) [file pone.0310795.s002.doc]

**Supporting information 2: Consent form in English and Hindi**

**Consent Form**

**Interventions to reduce stress among persons experiencing homelessness during the COVID-19 pandemic: a comparison of the stress reduction potential of Kuchipudi Dance and Bhagavad Gita**

I ____________, agree to participate in the abovementioned research project conducted by Laalithya Konduru, Simranjeet Singh Dahia, and Gargi Kothari-Speakman, who have discussed the research project with me.

I have received, read and kept a copy of the information letter/plain language statement. I have had the opportunity to ask questions about this research and I have received satisfactory answers. I understand the general purposes, risks and methods of this research.

I consent to participate in the research project and the following has been explained to me:

- the research may not be of direct benefit to me
- my participation is completely voluntary
- my right to withdraw from the study at any time without any implications to me
- the risks including any possible inconvenience, discomfort or harm as a consequence of my participation in the research project
- the steps that have been taken to minimise any possible risks
- what I am expected and required to do
- whom I should contact for any complaints with the research or the conduct of the research
- I am able to request a copy of the research findings and reports
- security and confidentiality of my personal information.

I consent to the publication of results from this study on the condition that my identity will not be revealed. In addition, ___________, my MOTHER/FATHER/SPOUSE, has provided oral consent for my participation on behalf of my family in my presence to the researchers mentioned above.

**Name:**

Signature:

Date:

WITNESSES (required in case participant cannot read or sign their name):

**1. Name:** _____________ 2. **Name:** _____________

Registration number: ____________ Registration number: ____________

Signature: ____________ Signature: ____________

Date: ______________ Date: ______________

सहमति प्रपत्र

कोविड-19 महामारी के दौरान बेघर होने का अनुभव करने वाले व्यक्तियों के बीच तनाव को कम करने के लिए परीक्षण: कुचिपुड़ी नृत्य और भगवद गीता की तनाव कम करने की क्षमता की तुलना

मैं ____ लालिथ्या कोंडूरू, सिमरनजीत सिंह दहिया और गार्गी कोठारी-स्पीकमैन द्वारा आयोजित उपर्युक्त शोध परियोजना में भाग लेने के लिए सहमत हूं| इन शोधकर्ताओं ने मेरे साथ अनुसंधान परियोजना पर चर्चा की है।

मैंने सूचना पत्र की एक प्रति प्राप्त की, पढ़ी और अपने पास रखी। मुझे इस शोध के बारे में सवाल पूछने का अवसर मिला है और मुझे संतोषजनक उत्तर मिले हैं। मैं इस शोध के सामान्य उद्देश्यों, जोखिमों और तरीकों को समझता हूं।

मैं अनुसंधान परियोजना में भाग लेने के लिए सहमति देता हूं और निम्नलिखित मुझे समझाया गया है:

• यह संभव है कि अनुसंधान मुझे सीधे लाभ नहीं पहुंचाएगा।

• मेरी भागीदारी पूरी तरह से स्वैच्छिक है

• मुझे किसी भी समय मेरे लिए किसी भी निहितार्थ के बिना अध्ययन से हटने का अधिकार है

• अनुसंधान परियोजना में मेरी भागीदारी के परिणामस्वरूप संभावित असुविधाएं या जोखिम

• किसी भी संभावित जोखिम को कम करने के लिए उठाए गए कदम

• मुझसे क्या उम्मीद की जाती है और मुझे क्या करने की आवश्यकता है

• अनुसंधान के संचालन के बारे में किसी भी शिकायत के लिए मुझे किससे संपर्क करना चाहिए

• मैं शोध निष्कर्षों और रिपोर्टों की एक प्रति का अनुरोध करने में सक्षम हूं।

• मेरी व्यक्तिगत जानकारी की सुरक्षा और गोपनीयता।

मैं इस अध्ययन के परिणामों के प्रकाशन के लिए इस शर्त पर सहमति देता हूं कि मेरी पहचान उजागर नहीं की जाएगी। इसके अलावा, मेरे माता / पिता / पति / पत्नी, _____, ने ऊपर उल्लिखित शोधकर्ताओं को मेरी उपस्थिति में मेरे परिवार की ओर से मेरी भागीदारी के लिए मौखिक सहमति प्रदान की है।

नाम: ___________________________________________________________________________

हस्ताक्षर: ________________________________________________________________________

दिनांक: _________________________________________________________________________

साक्षी (यदि प्रतिभागी अपना नाम नहीं पढ़ सकता या हस्ताक्षर नहीं कर सकता है):

1. नाम: 2. नाम:

पंजीकरण संख्या: पंजीकरण संख्या:

हस्ताक्षर: हस्ताक्षर:

दिनांक: दिनांक:
