# Supplementary material for: Registered report: The effectiveness of a Bhagavad Gita intervention to reduce psychological distress in homeless people—A randomised controlled trial
Source: PLoS One. 2024 Nov 5;19(11):e0310795. doi: 10.1371/journal.pone.0310795 (PMC11537408; doi:10.1371/journal.pone.0310795)
Supplement: Attachment — Submitted filename: Response_to_reviewers.docx [file pone.0310795.s003.docx]

**Response to Reviewers**

***Journal requirements:***

**When submitting your revision, we need you to address these additional requirements.**

**C1. Please ensure that your manuscript meets PLOS ONE's style requirements, including those for file naming. The PLOS ONE style templates can be found at** [**https://journals.plos.org/plosone/s/file?id=wjVg/PLOSOne_formatting_sample_main_body.pdf**](https://journals.plos.org/plosone/s/file?id=wjVg/PLOSOne_formatting_sample_main_body.pdf) **and** [**https://journals.plos.org/plosone/s/file?id=ba62/PLOSOne_formatting_sample_title_authors_affiliations.pdf**](https://journals.plos.org/plosone/s/file?id=ba62/PLOSOne_formatting_sample_title_authors_affiliations.pdf)

A1. We have diligently reviewed and revised our manuscript to ensure compliance with PLOS ONE's style requirements.

**C2. Your ethics statement should only appear in the Methods section of your manuscript. If your ethics statement is written in any section besides the Methods, please delete it from any other section.**

A2. We have carefully examined the placement of the ethics statement and ensured that it exclusively appears in the Methods section of the manuscript. Any instances of the ethics statement appearing in sections other than the Methods have been identified and promptly removed.

**C3. When completing the data availability statement of the submission form, you indicated that you will make your data available on acceptance. We strongly recommend all authors decide on a data sharing plan before acceptance, as the process can be lengthy and hold up publication timelines. Please note that, though access restrictions are acceptable now, your entire data will need to be made freely accessible if your manuscript is accepted for publication. This policy applies to all data except where public deposition would breach compliance with the protocol approved by your research ethics board. If you are unable to adhere to our open data policy, please kindly revise your statement to explain your reasoning and we will seek the editor's input on an exemption. Please be assured that, once you have provided your new statement, the assessment of your exemption will not hold up the peer review process.**

A3. We have made an error in saying that we will make the data available on acceptance. As this is only a protocol for a clinical trial, there is no data to share at present. We will make the raw data available when the study is completed, along with the Stage II manuscript containing the results of the trial. The raw data will be made available in a public repository such as Figshare. The submission form instructions for the data availability statement say that if *no pilot data are reported in the manuscript,* we must enter “*All relevant data from this study will be made available upon study completion”* as the data availability statement. This is what we have now entered as our data availability statement of the submission form.

***Reviewer #1:***

C1. **Lack of a clear and concise research question or objective statement.**

A1. We have made the central research question more obvious in the introduction. The revised text is found in Lines 113–115 in the clean copy of the manuscript.

Revised text: This study aims to bridge the knowledge gap concerning the effectiveness of Hindu RSIs in a previously unexplored population—PEH—by evaluating the impact of learning the Bhagavad Gita on reducing psychological distress among PEH. Engaging in Kuchipudi dance practice as a hobby will function as a positive control, while abstaining from stress-reducing activities will serve as the negative control. In other words, the central research question is, what is the impact of learning the Bhagavad Gita on reducing psychological distress among PEH, and how does this compare with the impact of engaging in Kuchipudi dance practice as a hobby? This multi-site parallel-group RCT seeks to comprehensively assess both short and long-term outcomes. A subgroup analysis will concentrate on the efficacy of learning the Bhagavad Gita among PEH who adhere to Hindu beliefs, investigating whether the Hindu RSI exerts a more pronounced influence on PEH embracing Hindu beliefs compared to those with other belief systems. Table 1 outlines the research inquiries guiding our study and presents potential interpretations of the anticipated results.

C2. **Insufficient background information and justification for the study.**

A2. We have provided additional background information to justify the study. The revised text is found in Lines 74–78 and Lines 87–95 in the clean copy of the manuscript.

Revised text: Engaging in hobbies has been shown to effectively reduce stress, foster social connections, and restore a sense of self among PEH.^6,7^ However, many of our clients have been unable to participate in recreational activities due to their daily struggles to earn a living, despite expressing a desire to engage in hobbies. Therefore, we sought a brief intervention that could enhance the coping abilities of PEH.

Findings from a previous study revealed that turning to religion emerged as a significant coping strategy for PEH, assisting them in managing the mental stress induced by the COVID-19 pandemic.^8^ Building on this crucial insight, we posit that a religious and spiritual Intervention (RSI) could offer valuable support to PEH.

A notable systematic review and meta-analysis focused on RSIs in randomized clinical trials (RCTs) has underscored the potential of RSIs in enhancing mental health outcomes; however, this review exclusively encompassed studies affiliated with Islamic, Christian, and Jewish traditions.^9^ The meta-analysis unveiled significant positive effects of RSIs on anxiety, general symptoms, and depressive symptoms. Nonetheless, due to the omission of any clinical trials focusing on the efficacy of Hindu RSIs or the impact of RSIs on individuals adhering to Hindu beliefs, a gap persists in understanding the benefits of Hindu RSIs, particularly among those embracing Hindu beliefs.

It is noteworthy that Hinduism is the most commonly practiced religion in India and there exist no empirical studies evaluating the effectiveness of Hindu RSIs. Therefore, tailoring an RSI to the Hindu context, especially in PEH, in the absence of empirical studies investigating the impact of Hindu RSIs, is a critical knowledge gap. Given the significant reliance on religious coping mechanisms among PEH^8^ and the dearth of research in this domain, our study aims to fill this void by specifically exploring the impact of learning the Bhagavad Gita—an essential Hindu scripture—as a potential RSI to alleviate psychological distress among PEH. This novel approach is informed not only by previous qualitative findings^8^ but also by the imperative to address the unique needs of this population through culturally relevant interventions.

The Bhagavad Gita offers practical guidance on navigating daily stressors.^10^ Within the realm of RSIs, recent studies offer insights into the potential relevance of the Bhagavad Gita's teachings for psychological well-being among adolescents^11^ and individuals coping with chronic illnesses^12^; however, these studies do not encompass a synthesis of empirical data to validate their findings. Recently, Das and Behura proposed that learning the Bhagavad Gita could improve the stress coping abilities of healthcare providers during the COVID-19 pandemic, drawing parallels between their experiences and those of war veterans; however, this study also lacked empirical substantiation.^13^

Considering that homelessness also poses significant challenges to human health, including mental health, and our organization’s volunteers had noted that a significant number of PEH had turned to spirituality as a coping mechanism to deal with the mental health challenges brought about by the COVID-19 pandemic, we hypothesized that imparting the teachings of the Bhagavad Gita as a one-time intervention could enhance the psychological resilience of PEH.

This study aims to bridge the knowledge gap concerning the effectiveness of Hindu RSIs in a previously unexplored population—PEH—by evaluating the impact of learning the Bhagavad Gita on reducing psychological distress among PEH.

Added Reference: [8] Konduru, L. & Das, N. Experiencing the COVID-19 pandemic as a homeless person in Chennai, India: An interpretative phenomenological analysis. PLOS One 18(11):e0295164 (2023). doi:10.1371/journal.pone.0295164.

C3. **Limited discussion of the existing literature and the gap that this research aims to address.**

A3. We believe that the gap that this study aims to address is now clear in the introduction after the changes detailed in A1 and A2. We are unable to find any RSIs in the Hindu context other than the ones we have already discussed. The good quality RSIs in other religious contexts were included the meta-analysis that we cited. We felt it prudent to discuss the results of the meta-analysis rather than the individual RCTs that were included in the meta-analysis as meta-analyses are a higher level of evidence than RCTs. We believe that the results of the meta-analysis are presented briefly but adequately. If there are any other studies that you would like us to discuss in the introduction, we request you to please specify these studies by author and date so that we can retrieve them and include a discussion about them.

C4. **Methods: Inadequate description of the study design, including the randomization process and sample size determination.**
A4. We have added 2 figures to clarify the Methods undertaken for this study in detail.

Regarding the concerns raised, our study incorporates a randomization process employing sealed envelopes selected by participants from a fishbowl, ensuring an unbiased group allocation (Lines 148–153 in the clean copy of the manuscript). The study design is mentioned in Lines 115–116 in the clean copy of the manuscript. Furthermore, the sample size determination is meticulously justified, considering alpha, beta, correlation, and minimum clinically significant difference (Lines 212–232 in the clean copy of the manuscript). We have enhanced the manuscript by adding a figure clarifying the study design.

*Fig. 2. Study flowchart*

C5. **Lack of details on the intervention itself, such as the specific content of the Bhagavad Gita intervention and the Kuchipudi dance practice.**

A5. In the section titled “Interventions”, we have added the specific information regarding the interventions. We are unable to provide a video of the choreography to make the content of the intervention even more explicit as the choreography is copyright protected. The content of the second chapter of the Bhagavad Gita is an important Hindu scripture and is widely available.

Revised text (Lines 156–168 in the clean copy of the manuscript): The participants in the Gita group will be imparted the teachings of the second chapter of the Bhagavad Gita (all verses) for 1 week, for 60 minutes per day. The teachings of the Bhagavad Gita will be imparted by a trained teacher and the second chapter has been chosen as it is said to contain the essence of the entire Bhagavad Gita.^14^ The participants in the Kuchipudi group will be taught the Kuchipudi dance choreography to a popular devotional song (“Sivashtakam”, choreographed by Padma Bhushan Dr. Vempati Chinna Satyam, song duration is approximately 10 min) for 60 minutes per day by a trained teacher for 1 week. Every day for 1 week, the participants in the Both group will be taught the Kuchipudi dance choreography to a popular devotional song (“Vandanamu Raghunandana”, choreographed by Kala Tilakam Dr. Sathyapriya Ramana, song duration is approximately 5 min), which is shorter in duration than the song used in the Kuchipudi group, for 30 minutes by a trained teacher, followed by imparting the teachings from selected verses of the second chapter of the Bhagavad Gita (Verse 40–72) for 30 minutes by a trained teacher. The participants in the control group will receive no intervention.

C6. **Absence of information on the data collection procedures, including the instruments used and the frequency of measurements. Insufficient explanation of the statistical analysis plan, including the primary and secondary outcomes and the methods for handling missing data.**

A6. We would like to clarify that the methods section provides detailed information on data collection procedures (Line 188–202 in clean copy of the manuscript), including the use of the original Kessler scale for psychological distress (Line 177–187 in clean copy of the manuscript). The questionnaire, filled by participants during scheduled sessions, will be sent by the participant to the project staff via WhatsApp.

The statistical analysis plan, involving generalized estimating equation for the primary outcomes and ANCOVA for subgroup analysis (Line 204–210 in clean copy of the manuscript), and sample size calculation (Line 211–232 in clean copy of the manuscript), is also outlined. The primary outcome, psychological distress measured by the K10, is clearly defined (Line 177–178 in clean copy of the manuscript). The plan for handling missing data, through either complete case analysis or multiple imputation, is specified (Line 243–263 in clean copy of the manuscript). If you require further details in specific areas, we are open to providing additional information.

C7. **Results: No presentation or discussion of any actual results or findings.
Lack of clarity on the interpretation of different outcomes and their significance.
Discussion: Limited discussion of the implications of the findings and their relevance to the existing literature. Inadequate exploration of the limitations of the study, such as potential biases or confounding factors. Insufficient consideration of the generalizability of the findings to other populations or settings.
Conclusion: Lack of a clear and concise summary of the main findings and their implications. Absence of specific recommendations or suggestions for future research.**

A7. As this is only a protocol, there are no results to present or discuss. We will conduct the study only after the acceptance of the stage 1 manuscript containing the protocol. The results, discussion, including the discussion of the limitations of the study that are not already discussed in this manuscript (Line 345–356 in clean copy of the manuscript), generalizability of the findings, and future directions of research, along with the conclusion will be presented in the Stage II manuscript where we will present the results of our study. We would like to reiterate that we have followed the SPIRIT checklist and provided all the information as required in the Stage 1 manuscript as per the checklist and PLOS ONE’s own guidelines for the Stage 1 registered report.

**C8. Overall, the document lacks crucial information in several sections, including the introduction, methods, results, discussion, and conclusion. It is important to provide more detailed and comprehensive descriptions of the study design, intervention, data collection, analysis plan, and interpretation of the findings. Additionally, addressing the limitations of the study and providing clear recommendations for future research would strengthen the manuscript.**

A8. Thank you for your valuable feedback. We have diligently addressed each concern, making appropriate revisions to the manuscript. The absence of results in this protocol has been clarified. Furthermore, to enhance clarity, transparency, and completeness, we have added a visual representation of the participant trajectory throughout the study in Fig 1. If there are any additional areas requiring attention or specific suggestions, please let us know, and we will gladly make further adjustments.

*Fig. 1. Participant journey: Navigating through the study*

***Reviewer #2:***

**C1. The study proposes a scientific investigation of a relevant topic with a clinical and social impact. The introduction sheds light on the subject clearly and concisely. The methodological design adequately followed international guidelines, especially randomization, allocation, and blinding, as far as possible. The intervention procedures are well described, clarifying what each intervention group will receive.
It would be interesting, if possible, to add other mental health outcomes, at least a satisfaction scale with the intervention. However, I imagine it would be challenging to carry out an extensive evaluation of this patient profile.**

A1. Thank you for your positive feedback and valuable suggestion. We acknowledge the importance of assessing satisfaction with the intervention. To address this, we are planning to conduct a focus group-based study in the future specifically designed to evaluate participant satisfaction with the interventions. We shall describe the same in the Stage II manuscript where we present the results of our study.

**C2. My final suggestion would be to explain what strategy will be used to conduct the interventions if it is not possible to recruit all participants on the same date in December 2023, since the estimated sample size is more than 200 participants. Will a new wave of recruitment be carried out? How will this be accomplished? I believe this information is important in the description the protocol.**

A2. As it is already January, and the paper is still under review, we have changed the timelines of the study in the manuscript as follows (Line 315–323 in clean copy of the manuscript):

Revised text: Engagement with PEHs begins in Week 1, followed by a 4-week recruitment period. Baseline measurements will be conducted in Week 5, interventions in Weeks 6–7, and post-intervention assessments in Weeks 8, 12, and 56. Interim analysis shall be conducted in Weeks 13–19, and results will be published as the Stage II of this registered report. Final analysis will be conducted in Weeks 57–61, and results shall be published as a brief report. Conferences, meetings, workshops, and other community engagement events to disseminate the results of the study will be ongoing indefinitely, starting from the date of publication of the interim analysis as a registered report. The detailed timeline is shown in Figure 3.

*Fig. 3. Timeline of the study*

Samanjasa Foundation operates in four distinct cities, providing assistance to over 500 PEH in Dhanbad and Chennai, the chosen locations for our study. The organization's extensive outreach and ongoing communication with clients have generated significant interest, resulting in a higher number of inquiries than needed for the study. Given this enthusiastic response and the dedicated efforts of our volunteers, we are confident in our ability to swiftly recruit the required participants within the planned four-week timeframe. However, in case we are unable to recruit the required number of participants, we will increase the recruitment period by another 4 weeks and open the recruitment to the remaining locations where Samanjasa Foundation operates, viz., Hyderabad and Bhubhaneswar, India. We have added this information to the section titled “Plan for handling missing data and protocol deviations” (Line 243–248 in clean copy of the manuscript).

Revised text: In case we are unable to recruit the required number of participants for the study from only Chennai and Dhanbad during the envisaged recruitment period, we will open the recruitment to the remaining locations that Samanjasa Foundation operates in, i.e., Hyderabad and Bhubaneshwar, India, and extend the recruitment period by another 4 weeks. Consequently, the timelines for the baseline measurements, interventions, and all post-intervention measurements will be delayed by 4 weeks.
