# Supplementary material for: Registered report: The effectiveness of a Bhagavad Gita intervention to reduce psychological distress in homeless people—A randomised controlled trial
Source: PLoS One. 2024 Nov 5;19(11):e0310795. doi: 10.1371/journal.pone.0310795 (PMC11537408; doi:10.1371/journal.pone.0310795)
Supplement: Attachment — Submitted filename: Response to reviewers.docx [file pone.0310795.s004.docx]

PONE-D-23-29874R1

Registered Report: The Effectiveness of a Bhagavad Gita Intervention to Reduce Psychological Distress in Homeless People—A Randomised Controlled Trial

**Response to Reviewers**

Journal Requirements:

Please review your reference list to ensure that it is complete and correct. If you have cited papers that have been retracted, please include the rationale for doing so in the manuscript text, or remove these references and replace them with relevant current references. Any changes to the reference list should be mentioned in the rebuttal letter that accompanies your revised manuscript. If you need to cite a retracted article, indicate the article’s retracted status in the References list and also include a citation and full reference for the retraction notice.

We can confirm that we have not cited any retracted papers. We noticed that we did not cite two papers pertaining to our interventions; so, we have added these citations. These are now references 6 and 7 in the revised manuscript. We have renumbered all the citations subsequently. These are the only changes to the reference list.


Reviewers' comments:

Reviewer's Responses to Questions

**Comments to the Author**

Reviewer #2: Thank you for addressing all comments in a satisfatory and careful manner. The study is well planned and will be relevant to incentivate objective and low-cost interventions to promote health for vulnerable population. Good luck with the progress of the study.

Thank you for taking the time to review our paper and for the encouragement.

Reviewer #3: This an interesting study. Assessing various interventions on the mental well being outcomes in the post Pandemic era.

Thank you for the positive assessment of our manuscript.

Some Minor comments for the author.
1. For Randomisation more detail is required relating to the randomisation schedule generation. The authors state the allocation ratio. But most importantly who created the schedule?

We have added the details regarding the randomization as follows (Lines 150-155):

“The participant ID will be generated in a random order by an independent statistician using a pseudo-random number generator script in Python 3.9.13. Group assignment will be generated in sequence by the independent statistician; the participant ID generated first will be associated with the Gita group, the second with the Kuchipudi group, the third with the Both group, the fourth with the Control group, the fifth with the Gita group, and so on. The project staff will fill and seal the envelopes with the respective contents.”

2. Commend the authors for ensuring allocation concealment was achieved.

We thank the reviewer for the kind words.

3. How would contamination be monitored? I.e individual randomisation , do you have people in the same community, meaning risk of individuals communicating.

We would like to thank you for this question; we had not considered this earlier, but thanks to your question, we have given this some thought and have added the following information to the manuscript (Lines 159-166):

“To monitor and minimize the risk of contamination, especially given the potential for participants within the same community to communicate, the project staff will send weekly text messages to the participants regarding not sharing details about the intervention with anyone, including their family members. Additionally, the project staff will randomly contact three participants from each group via a phone call every two months and ask them if they had heard anything about the interventions in the other study groups. Furthermore, during community events and interactions, case workers will be asked to assess the level of knowledge regarding the intervention among the PEH.”

4. Will there be a plan for drafting and covering methodological aspects in the analysis plan?

We have added a separate section titled “Statistical analysis plan” to the revised manuscript. The content of the section is as follows (Lines 287-355):

***“Statistical analysis plan (SAP)***

This SAP outlines the procedures for data cleaning, handling missing data, conducting primary and subgroup analyses, and performing sensitivity analyses to ensure the robustness of the findings.

*Data cleaning*

1. Data Entry and Validation: All data will be entered into a secure electronic database. Double data entry will be performed to ensure accuracy. Discrepancies will be resolved by consulting the original data source.
2. Consistency Checks: Logical checks will be conducted to identify any inconsistencies (e.g., age values outside the expected range). Outliers will be flagged and verified with the original data.
3. Descriptive Summaries: Baseline characteristics of participants will be summarized using means and standard deviations for continuous variables and frequencies and percentages for categorical variables.

*Handling of missing data*

1. Identification: Missing data will be identified and categorized (e.g., missing completely at random, missing at random, or not missing at random).
2. Imputation: Multiple imputation methods will be used to handle missing data, particularly if the proportion of missing data exceeds 5%. Sensitivity analyses will be conducted to compare the results from imputed and non-imputed datasets.
3. Documentation: The extent and nature of missing data will be documented and reported.

*Primary analysis*

1. GEE Framework: The primary outcome, psychological distress as measured by the K10 scale, will be analyzed using the GEE framework. Robust standard errors will be used to account for correlated data due to repeated measures. An exchangeable correlation structure will be assumed.
2. Primary Comparisons: Comparisons will be made between each intervention group (Gita, Kuchipudi, and Both) and the control group. Within each intervention group, comparisons over time (pre-intervention, immediate post-intervention, 40 days post-intervention, and 1-year post-intervention) will be performed.
3. Pairwise Post-Hoc Comparisons: Pairwise post-hoc comparisons will be conducted following significant GEE results to identify specific group differences. The Bonferroni correction will be applied to control for the risk of type I error in these multiple comparisons.

*Subgroup analyses*

1. ANCOVA/Non-Parametric ANCOVA: Subgroup analyses will be conducted using ANCOVA or non-parametric ANCOVA depending on the normality of the data. Subgroups may include stratification by age, educational attainment, religion, and employment status.
2. Multiple Comparisons: To address the risk of type I error due to multiple comparisons, appropriate statistical corrections will be applied, which may include the Bonferroni correction, Holm's adjustment, or the Benjamini-Hochberg procedure.

*Sensitivity analyses*

1. Robustness Checks: Sensitivity analyses will be performed to assess the robustness of the primary findings. Analyses will include different imputation methods for missing data and alternative statistical models (e.g., mixed-effects models).
2. Assumption Testing: Assumptions of the GEE and ANCOVA models will be tested, including the assumption of normality and homogeneity of variances. Alternative models or transformations will be used if assumptions are violated.
3. Exclusion of Outliers: Sensitivity analyses will be conducted with and without outliers to determine their impact on the study results.

*Adjustment and interpretation based on contamination checks*

If contamination is detected, we will take the following steps to adjust or interpret the study results:

1. Quantification of Contamination: We will quantify the extent of contamination by calculating the percentage of participants who have been exposed to details about interventions other than their own.
2. Statistical Adjustment: We will use statistical techniques to adjust for contamination. For instance, we might include contamination status as a covariate in our regression models to control for its potential impact on the study outcomes.
3. Sensitivity Analyses: We will conduct sensitivity analyses to assess the robustness of our findings. This will involve comparing the results of the main analysis with those obtained after excluding contaminated participants or adjusting for contamination.
4. Interpretation of Results: In the discussion section, we will interpret our findings in light of the contamination checks. We will acknowledge the presence of contamination, describe its potential impact on the study outcomes, and discuss how our adjustments have accounted for it.

This SAP will be reviewed and updated as necessary throughout the study to ensure the robustness and integrity of the analyses and findings.”

5. Statistical analysis methods- mention descriptive summaries of baseline characteristics and also that results will be reported in accordance with CONSORT.

In accordance with your comment, we have added the following text to the data analysis subsection:

“All results will be reported in accordance with the CONSORT guidelines to ensure comprehensive and transparent reporting of the trial's outcomes. Descriptive summaries of baseline characteristics will be presented to provide an overview of the study population.”

6. They are four arms, in the methods section can the authors explicitly state the comparisons, do the authors need to consider multiple comparisons?

We have explicitly stated the comparisons, including multiple comparisons, in the statistical analysis section mentioned above (response to comment #4). In addition, we have amended the data analysis subsection as follows (the yellow highlights are the changed parts; Lines 220-233):

“The comparisons will be between each intervention group and the control group, as well as within each intervention group over time. The repeated measures data will be analysed using the generalised estimating equation (GEE) framework. For the analysis of the primary outcome using the GEE framework, robust standard errors will be used to account for correlated data. Pairwise post-hoc comparisons will be conducted to identify specific group differences. To control for the risk of type I error in these multiple comparisons, the Bonferroni correction will be applied. Subgroup analysis will be conducted using analysis of covariance (ANCOVA) or non-parametric ANCOVA^19^ depending on the normality of the data; this may include stratification by age, educational attainment, religion, and employment status. Multiple comparisons will also be addressed using appropriate statistical corrections. These may include the Bonferroni correction, Holm's adjustment, or the Benjamini-Hochberg procedure, depending on the specific comparisons and the number of tests conducted. The data analysis will be conducted using Python 3.9.13. Statistical significance will be set at a two-tailed p < 0.05.”

We have sincerely hope that the changes made to the manuscript are to your satisfaction.
