# Supplementary material for: Registered report: The effectiveness of a Bhagavad Gita intervention to reduce psychological distress in homeless people—A randomised controlled trial
Source: PLoS One. 2024 Nov 5;19(11):e0310795. doi: 10.1371/journal.pone.0310795 (PMC11537408; doi:10.1371/journal.pone.0310795)
Supplement: Attachment — Submitted filename: Response to reviewers.docx [file pone.0310795.s005.docx]

PONE-D-23-29874R2

Registered Report: The Effectiveness of a Bhagavad Gita Intervention to Reduce Psychological Distress in Homeless People—A Randomised Controlled Trial

**Response to Reviewers**

***Journal Requirements:***

Please review your reference list to ensure that it is complete and correct. If you have cited papers that have been retracted, please include the rationale for doing so in the manuscript text, or remove these references and replace them with relevant current references. Any changes to the reference list should be mentioned in the rebuttal letter that accompanies your revised manuscript. If you need to cite a retracted article, indicate the article’s retracted status in the References list and also include a citation and full reference for the retraction notice.

We can confirm that we have not cited any retracted papers. The reference list is complete and correct. There are no changes to the reference list.


***Comments to the Author:***

While nearly ready for publication, we do have an additional request- In the introduction, you state that "Subgroup analysis will concentrate on the efficacy of learning the Bhagavad Gita among PEH who adhere to Hindu beliefs, investigating whether the Hindu RSI exerts a more pronounced influence on PEH embracing Hindu beliefs compared to those with other belief systems". However, in my reading of the manuscript, I don't think this is captured in the methodology. Therefore, please either update your methodology to include a description of this investigation, or remove this section from the introduction.

We have removed the section from the introduction.
